# Necroptosis: a regulated inflammatory mode of cell death

**DOI:** 10.1186/s12974-018-1235-0

**Published:** 2018-07-06

**Authors:** Yogesh K. Dhuriya, Divakar Sharma

**Affiliations:** 1 Developmental Toxicology Laboratory, Systems Toxicology and Health Risk Assessment Group, CSIR-Indian Institute of Toxicology Research (CSIR-IITR), Vishvigyan Bhawan; 31, Mahatma Gandhi Marg, Lucknow, 226001 India; 2 Academy of Scientific and Innovative Research (AcSIR) Lucknow Campus, Lucknow, India; 3 Department of Biochemistry, National JALMA Institute for Leprosy and Other Mycobacterial Diseases, Tajganj, Agra, India; 4 Interdisciplinary Biotechnology Unit, Aligarh Muslim University, Aligarh, 202002 India

**Keywords:** Necroptosis, Inflammation, Neurodegenerative disease, RIPK3, MLKL

## Abstract

Programmed cell death has a vital role in embryonic development and tissue homeostasis. Necroptosis is an alternative mode of regulated cell death mimicking features of apoptosis and necrosis. Necroptosis requires protein RIPK3 (previously well recognized as regulator of inflammation, cell survival, and disease) and its substrate MLKL, the crucial players of this pathway. Necroptosis is induced by toll-like receptor, death receptor, interferon, and some other mediators. Shreds of evidence based on a mouse model reveals that deregulation of necroptosis has been found to be associated with pathological conditions like cancer, neurodegenerative diseases, and inflammatory diseases. In this timeline article, we are discussing the molecular mechanisms of necroptosis and its relevance to diseases.

## Background

Cell demise and its survival are the fundamental features of metazoans to maintain the tissue homeostasis. On morphological basis, cell death is achieved by apoptosis, necrosis, and autophagy [1]. A plethora of studies has been performed on apoptosis and autophagy which reveals a clear picture of molecular mechanisms of apoptosis and autophagy and is recognized as a highly regulated process. Hence, apoptosis and autophagy are regarded as “programmed cell death” while necrosis is considered as “unprogrammed” due to deregulated activity. Decades ago, a novel type of cell death was reported where apoptotic pathway was inhibited, which exhibited morphological features of both apoptosis and necrosis and hence was termed as necroptosis [2]. Interestingly, necroptosis is inhibited by Necrostatin-1 (Nec-1) by inhibiting the activity of receptor-interacting protein kinase 1 (RIPK1), which suggests that it is a well-regulated process or programmed necrosis. Later studies on morphological features of necroptosis confirmed that it is the unregulated necrotic death, stimulated by the secretion of cytokines/chemokines resulting in inflammation [3]. Inflammation involves a series of reactions in response to pathogen-infected cells resulting in the elimination of infected cells as well as wound healing.

Necroptosis is a regulated necrosis mediated by death receptors [4]. This form of necrosis works against pathogen-mediated infections, morphologically characterized by cell swelling followed by rupturing of plasma membrane. It is well known that involvement of receptor like Fas, TNF, and TRAIL can lead to cell death through the recruitment of caspase-8 leading to initiation of extrinsic apoptotic pathway [5]. A plethora of evidences has shown that inhibition of caspase-8 molecule shift extrinsic apoptosis to necrosis mode of cell death due to activation of RIPK3 and MLKL [6–10]. Hence, it is an alternative mode of cell death when caspase-8-dependent apoptotic pathway is blocked. Initiation of necroptosis is mediated by immune ligands including Fas, TNF, and LPS leading to activation of RIPK3 which further activates the MLKL by phosphorylation [10]. Phosphorylated MLKL translocates into the inner leaflet of the plasma membrane and disturbs the integrity of the cell [11–13]. Although RIPK3 and MLKL is necessary for programmed cell death [10, 14, 15], necroptosis is confined to certain types of tissue that express RIPK3/MLKL. In normal circumstances, caspase-8 molecule activates apoptosis by blocking the necroptosis and by cleaving RIPK1 and CYLD [16–18]. Classical necrosis leads to increased secretion of cytokines and decreased secretion of damaged associated molecular pattern (DAMP—endogenous molecules released in response to tissue damage) in contrast to necroptosis. Tumor necrosis factor (TNF) promotes the inflammatory cytokine synthesis in most of cell types resulting in various inflammatory diseases like Crohn’s disease, psoriasis, bowel disease, and rheumatoid arthritis [19, 20] and regulates production of chemokines and cytokines as primary outcomes of TNF stimulation [21]. Shifting of TNF-mediated response into programmed necrosis is not simply shifting of inflammation to necroptosis; it represents shifting of robust inflammatory response into necroptosis which terminates earlier in contrast to classical pro-inflammatory response. There are two basic conditions for necroptosis: (1) cells must express RIPK3 and (2) inhibition of caspase-8 molecule. Recent study of Moriwaki and Chan [22] has shown that MLKL rather than RIPK3 decides whether cell undergoes apoptosis or necroptosis which further suggests that expression of MLKL is necessary for induction of necroptosis using RIPK3 T231A/S232A mutant-expressing cells. Numerous in vitro studies have reported that inhibition of caspase-8 molecules resulted in activation of RIPK3, a key player of necroptosis [6, 7, 23]. Duprez et al. [24] have shown that caspase-8 inhibition might not always be mandatory to trigger the in vivo necroptosis.

## Molecular mechanism of necroptosis

**Fig. 1:**
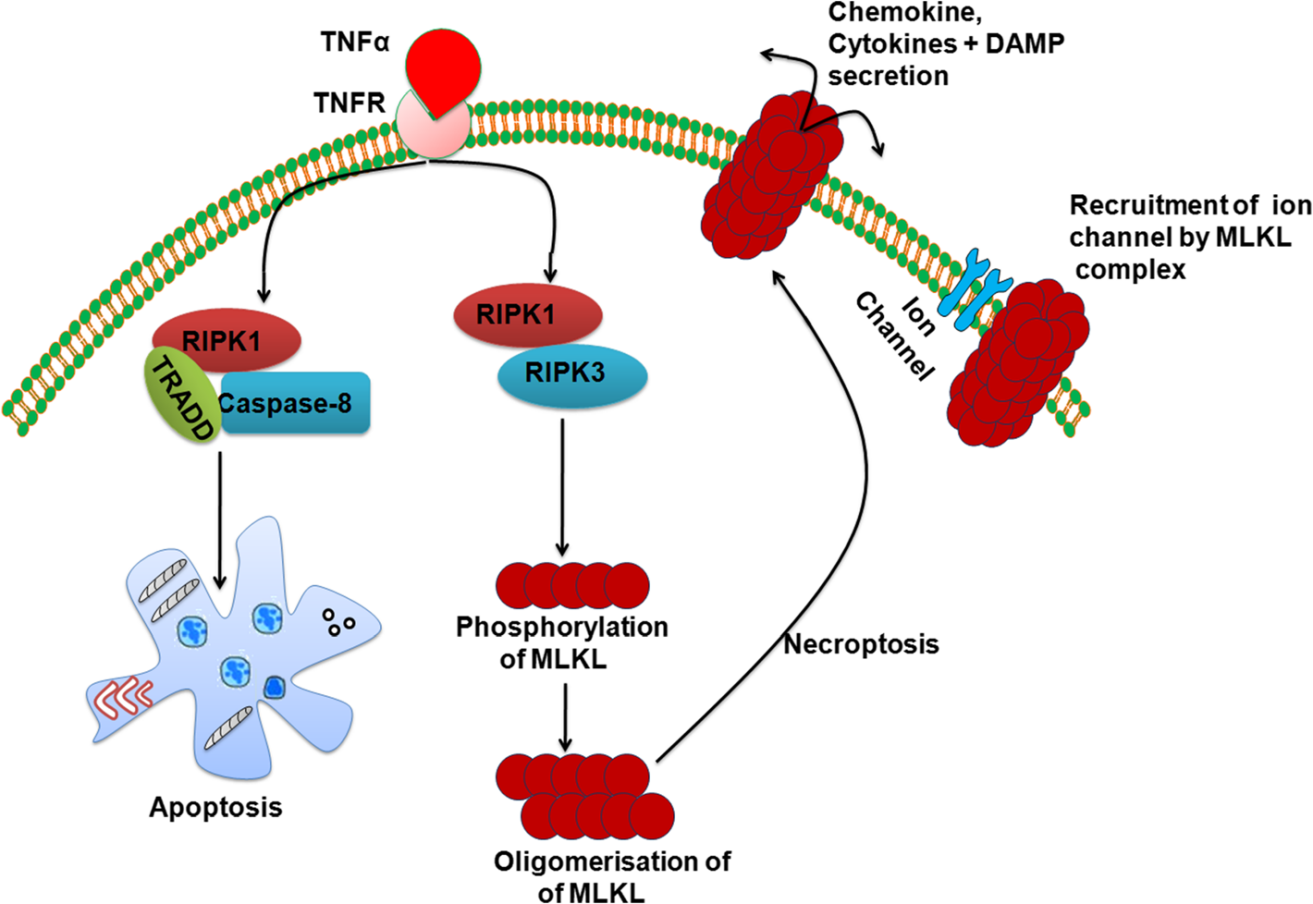
Molecular mechanism of apoptosis and necroptosis. Death receptor mediates both extrinsic apoptosis as well as necroptosis; RIPK1 plays a key role in apoptosis and necroptosis. Activation of caspase-8 drives the pathway towards apoptosis while its inhibition leading to necroptosis. During necroptosis, RIPK1 and RIPK3 interact with each other resulting in the formation of functional heterodimer complex; this complex promotes oligomerization of MLKL by phosphorylating it. Oligomeric form of MLKL translocates towards the plasma membrane from cytosol resulting in the formation of the pore, causing an inflammatory response. In spite of pore formation, MLKL also mediates its effect after interacting with ion channels

Recent studies have been focused on TNFα, RIPK3, and caspase-8 to understand the molecular mechanism of necroptosis. Necroptosis can be initiated by TNF superfamily receptors, toll-like receptors (TLR3 and TLR4), and interferon receptors while TNFR1-mediated necroptosis is well characterized. Base on the driving factors, necroptosis is classified into three categories: (1) Extrinsic necroptosis is stimulated by TNFα, (2) Intrinsic necroptosis is stimulated by reactive oxygen species (ROS), and (3) Ischemia mediated intrinsic necroptosis. TNFα-mediated necroptosis is a classical necroptosis which binds with complementary receptor leading to formation of short-lived membrane signaling complex (known as complex I) containing TRADD, FADD, RIPK1, TRAF2/TRAF5, and cIAP1/cIAP2. TRADD is an adaptor molecule recruit RIPK1 to TNFR1 [25, 26]. Subsequently, cIAPs and TRAF2/3/5 are recruited to complex I [27]. On activation, cIAP1/2 and TRAF2/5 mediate ubiquitination of RIPK1 which resulted in formation of stable complex I and initiate alternative pathway that culminates with cell survival pathway including NF-кB- and MAPK-mediated pathway [28]. NF-кB signaling plays a key role in counteracting the cytotoxic effect of TNFα, and prosurvival effect of NF-кB is mediated by cIAP1/2 and cFLIP_L_ (cellular FLICE-like inhibitory protein) [29, 30]. Hence, complex I is a crucial checkpoint for cell survival and necroptosis [31]. Normally, apoptosis is inhibited by formation of a heterodimer of caspase-8 and cFLIP_L_ leading to inactivation of caspase-8. Caspase-8 induces the exogenous apoptosis and deactivates the necroptosis by inhibiting the activity of RIPK3 and RIPK1. Elimination or inhibition of caspase-8 leads to activation of RIPK1 through deubiquitination mediated by cylindromatosis (CYLD) [16, 32] thus destabilizing the complex I. Removal of ubiquitin chain from RIPK1 leads to its interaction with FADD, TRADD, RIPK3, and caspase-8 which further resulted in formation of complex II. RIPK1 interacts with RIPK3 through receptor homology domain (RHD) leading to formation of necrosome which further initiates the downstream signaling resulting in necroptosis [33]. Although both RIPK3 and RIPK1 are necessary for induction of necroptosis, RIPK3 can alone promote necroptosis when it is overexpressed in cells [34]. On activation, RIPK3 phosphorylates the pseudokinase MLKL (mixed lineage kinase domain-like protein) which plays a key role in induction of necroptosis. MLKL acts in two ways: (1) either it acts as platform in plasma membrane for recruitment of Na^+^ ion or Ca^++^ channels or (2) promotes the pore formation in plasma membrane by interacting with amino terminal of phosphotidyl inositol phosphate (Fig. 1) [10, 35]. Wang et al. [36] reported the role of mitochondrial molecule PGAM (phosphatase phosphoglycerate mutase)-5 in necroptosis while the role of PGAM-5 is still controversial as many studies have shown that even complete depletion of the mitochondria did not influence the necroptosis processes [37].

## Non-classical necrosome

In classical necroptosis, necrosome complex is formed due to interaction between RIPK3 and RIPK1 through the RHIM domain. Phosphorylation of these proteins at their kinase domain promotes RHIM-mediated interactions of both proteins which results in formation of amyloid-like filamentous signaling complex [38, 39] and culminates with necroptosis. In spite of these proteins, many other proteins like TLR3/4, TRIF, and DAI (DNA activator of interferon) also have RHIM domain; hence, they can also form the necrosome which is considered as non-classical necrosome. Further, proteins sharing the RHIM domain may share functions in cell death signaling, innate immune signaling, or both. TLR3 and TLR4 also initiate the necroptosis mediated by TRIF and RIPK3 [40, 41]. Like RIPK3 and RIPK1, TRIF is also a cleavage substrate for caspase-8. TRIF-dependent necroptosis also requires interaction with RIPK3 through the RHIM domain while the role of RIPK1 is not clear. Several studies have reported that inhibition of RIPK1 in TLR3-mediated necroptosis does not impair this process [41]. Unlike RIPK, TRIF does not possess protein kinase activity which indicates that the mechanism by which TRIF activates the RIPK3 is different from RIPK1-mediated activation of RIPK3 and so this pathway is considered as non-classical necroptosis.

## Role of phospho-acceptor sites in RIPK1 and RIPK3

Phosphorylation plays a major role in controlling the activity of proteins. Mass spectrometry analysis has shown that RIPK1 and RIPK3 have multiple sites for phosphorylation on N-terminal kinase domain. Interestingly, expression of the truncated form of these proteins lacking N-terminal kinase domain resulted in the formation of amyloid fibrils [3]. Phospho-acceptor sites in RIPK1 are ser89 and ser161. Addition of phosphate group on ser89 inhibits the activity of RIPK1; hence, this site is involved in regulation of RIPK1 activity during necroptosis. McQuade et al. [42] have reported that substitution of serine with glutamate (S89D-RIPK1) resulted in reduced activity of RIPK1 while in the case of S89A-RIPK1, kinase activity of RIPK1 is increased in contrast to wild-type RIPK1. Structural analysis of RIPK1 with B-Raf suggested that Ser161 is necessary for pro-necrotic activity of RIPK1. Pro-necrotic activity of RIPK1 has been found to reduce the substitution of serine with alanine (S161A-RIPK1) [3, 42]. In spite of this, Ser321 is also important for induction of RIPK1-mediated necroptosis, [43] and in addition, Newton et al. [44] have shown that autophosphorylation of Ser166 of RIPK1 also mediates the induction of necroptosis. The two major phosphorylation sites in RIPK3 are ser204 and ser232. Sequence homology experiments have shown that ser204 in mouse (ser199 in human) is conserved in different species. Experimental studies reported that substitution of ser at 204 (S204A-RIPK3) resulted in inhibition of necroptosis [14, 42]. In spite of this, ser232 plays a key role in the recruitment of MLKL; phosphomimetic studies have shown that ser232 did not control kinase activity of RIPK3 while it disrupts the binding surface for MLKL [10, 42]. Besides these, ser227 also mediates MLKL binding to RIPK3; phosphomimetic mutant at ser227 inhibits RIPK3-mediated necroptosis [10, 42]. These studies suggested that phosphorylation at ser227 allows permissive conformation in RIPK3 to interact with MLKL. Although, RIPK1 is an upstream kinase which activates RIPK3 but RIPK3 and can induce the necroptosis independently of RIPK1. Some studies have also shown that inducible dimerization of RIPK3 initiates the MLKL-dependent necroptosis [45–49] and these results suggested that RIPK1 promotes nucleation events for RIPK3 oligomerization.

## Necroptosis and its role in inflammation

**Fig. 2:**
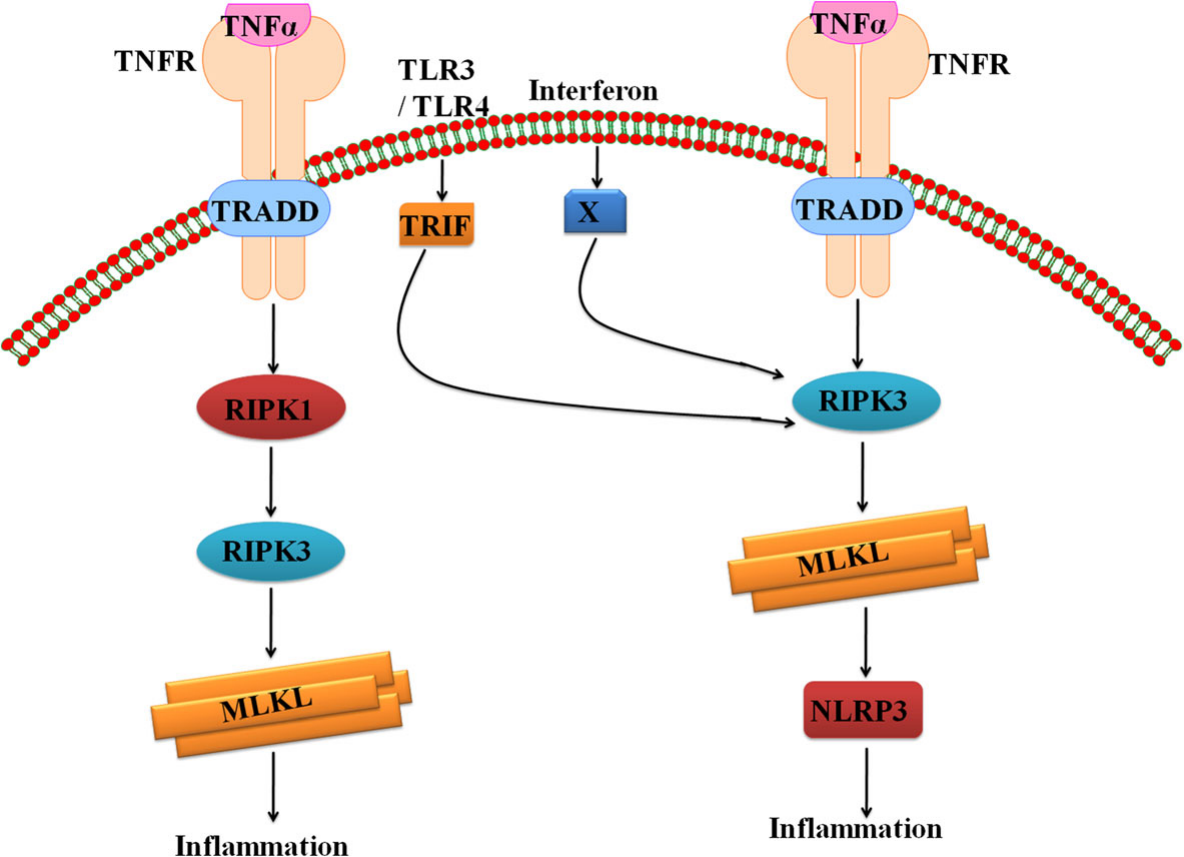
RIPK-dependent inflammation. RIPK3 kinase activity, critical for oligomerization of MLKL that culminates with inflammation. Activation of RIPK3 is mediated by RIPK1 as well as other mediators like TLR3/TLR4 and interferon

In case of apoptosis, secretion of cytokines is absent or very less, while during necroptosis, it is a primal event leading to robust inflammation. However, release of DAMP from cells is the primary way by which RIPK3 stimulates the inflammatory response after insertion of MLKL. Recent studies have reported that RIPK3 also directly activates the formation of inflammasome which is formed in response to cellular stress or microbial infection to activate caspase-1 and caspase-11. Further, caspase-1 cleaves IL-1β into mature form [50] and this activation occurs via two distinct RIPK3-dependent pathways: one mediated by caspase-8 and the other one mediated by NLPR3 (NOD, LRR, and pyrin) and leads to inflammasome formation [51]. A number of studies have shown that RIPK3 facilitates cytokine production and activation of inflammasome which is mediated by lipopolysaccharide (LPS) [52–56]. Several studies have reported that MLKL is essential for RIPK3-dependent inflammation (Fig. 2) while few studies reported that ablation of MLKL does not affect the RIPK3-dependent activation of NLPR3, IL-1β maturation, or cytokine production [57–59]. However, it is unknown how RIPK3 activates NLPR3-mediated formation of inflammasome with or without the involvement of MLKL. Some models explain that RIPK3 also acts as scaffold to recruit complex containing RIPK1, FADD, and caspase-8 [51, 52, 56, 59, 60]. In this condition, caspase-8 promotes the maturation of naive IL-1β by an unknown mechanism or activates the caspase-1 within NLPR3 inflammasome. However, some studies have shown that caspase-8 also plays an inhibitory role by preventing RIPK3-MLKL-mediated assembly of NLPR3 [53].

In spite of RIPK3, RIPK1 also induces cytokine production independent of RIPK3. In some models, RIPK1 acts as scaffolds especially during TNF-mediated NF-κB and JNK activation leading to cytokine productions [61–63]. Recently, it was shown that RIPK1 is crucial in increasing the level of circulating IL-1α for activation of NF-κB, FOS, and ERK following TLR4 activation [64], for secretion of TNF of TNF-treated cells in an autocrine fashion [65] and also for the induction of spontaneous inflammatory disease in SHIP (defective SH2 domain-containing inositol 5′-phosphatase 1) defective mice [66]. The release of DAMPs either from dying cells or by RIPK1–RIPK3 inflammasome-dependent or RIPK1–RIPK3 inflammasome-independent pathways varies depending on the cellular environment.

## Necroptosis: infectious and non-infectious diseases

Necroptosis plays a key role in the production of cytokine driven by TNF on pathogen infection. TNF is a major driver of bacterial infection suggesting that necroptosis may also appear to be a pro-inflammatory factor in bacterial infection-induced inflammation. *Escherichia coli*-expressed NleB1 protein (pathogenicity effector protein) inhibits apoptosis and necroptosis by modifying arginine residue in proteins containing death domains such as FADD and RIPK1 [67, 68]. NleB1 (pathogenecity effector protein having N-acetylglucosamine transferase activity)-deficient bacteria (*E*. *coli*) are unable to colonize in the intestine of the host which suggests that bacterium-induced cell death protects the host organism. In addition to this, RIPK3 deficiency in combination with caspase-8 or FADD leads to increased susceptibility to *Yersinia* infection [13, 69]. Several studies have shown that many bacteria stimulate RIPK3-mediated necroptosis [70–73]. Consistent with this, RIPK3-dependent necroptosis and TNF expression have been observed in tissues infected with *Mycobacterium tuberculosis* [72], further suggesting that necroptosis plays a key role in bacterial-induced chronic inflammation. Roca and Ramakrishnan [72] also showed that RIPK1/RIPK3 are both essential to stimulate TNF-dependent generation of reactive oxygen species (ROS) in tuberculosis infection. Experimental studies have also shown that upregulation of RIPK3 and MLKL detected in alcoholic and drug-induced liver injury suggests that necroptosis is also involved in sterile inflammation. Application of Necrostatin (Nec)-1 or depletion of RIPK3 protects liver cells from these types of injuries [74]. Parasitic diseases like leishmaniasis and malaria generally caused hemolysis, anemia, and sometimes bleeding. These result due to rupturing of red blood corpuscles (RBCs) leading to release of hemoglobin (Hb) into circulation; heme is produced on oxidation of Hb leading to initiation of the Fenton reaction and culminates with generation of ROS. Heme is also responsible for direct activation of TLR4, leading to autocrine secretion of ROS and TNF, and they activate the RIPK1/3-dependent necroptosis in a synergistic manner [75].

In spite of this, RIPK/MLKL-mediated necroptosis also plays a key role in destructive inflammation during viral infection. Viruses use the signaling pathways of the host to potentiate infection such as anti-apoptotic proteins encoded by viruses which increase its ability to replicate inside the host cell. Upton et al. [76] have shown that viral (mouse cytomegalovirus and M45-encoded viral inhibitor of RIPK activation) encoding protein containing the RHIM domain interacts with RIPK1 and RIPK3 and inhibits virus-induced cell death. Viral inhibitor of RIPK activation (vRIA) disrupts the binding of RIPK3 with DNA-dependent activator of IRFs (DAI) which results in suppression of cytomegalovirus-mediated necroptosis [77] while human cytomegalovirus-encoded different protein (IE1—immediate early gene 1) which does not disrupt the binding of RIPK3 with DAI; it acts by inhibiting signaling downstream of MLKL [78]. Experimental studies carried out on mice lacking RIPK3 exhibit impaired virus-induced necroptosis and increased sensitivity to viral infection [8, 13, 77, 79, 80]. Regulation of necroptosis by viruses appears to be detrimental to the host under some circumstances such as in the case of HIV infection which induces necroptosis in immune cells required for infection control. The rate of necroptosis was increased in HIV-infected T cells which were correlated with decreased caspase-8 activity [81] and higher sensitivity to TNF-mediated cell death [82]. Several studies have reported the role of necroptosis in multiple tissues in ischemia-reperfusion condition [83–85]. In addition to this, fewer necrotic areas and less pro-inflammatory cytokine expression in active necroptosis lesson has been found in RIPK3-deficient mice; they are also more resistant to the development of atherosclerosis [86, 87].

## Necroptosis and neurodegenerative disease

Necroptosis was characterized initially in ischemic brain. Several lines of evidences have reported that necroptosis not only caused pathogenesis of neurodegenerative diseases such as Parkinson’s disease [88], amyotrophic lateral sclerosis [89, 90], and multiple sclerosis (MS) [91] but is also involved in other neurodegenerative conditions including spinal cord injury [92, 93] and retinal degeneration [63, 94, 95]. A secondary pathological feature in the patient of spinal cord injury is chronic inflammation, astrogliosis, and cavity formation [96]. Some studies have shown that application of Nec-1 has a protective effect in spinal cord injury (SCI) [14, 97]. A recent study has reported that expression of RIPK3 and phosphorylated MLKL increased in reactive astrocytes and microglia after SCI [92, 98]. M1 microglia induced TLR/myeloid differentiation signaling-dependent necroptosis leading to cell death of reactive astrocytes which line the spinal cavity [92], and microglia plays a key role during chronic inflammation post-SCI [98]. Microglial-mediated chronic inflammation further raises questions how programmed necrosis regulates the chronic inflammation after SCI. Multiple sclerosis is an autoimmune disease of the brain characterized by demyelination and chronic inflammation. Ofengeim et al. [91] have reported that TNFα induces the death of oligodendrocytes in a RIPK1/3-dependent manner. Increased level of TNF-α, IL-1β, and RIPK3 in microglia and neurons in the mouse model mimics the characteristic of Gaucher’s disease, a metabolic disorder of the brain. Amyotrophic lateral sclerosis (ALS), a well-known motor neuron degenerative disease, is characterized by inflammation which is a hallmark of ALS. Re et al. [99] have shown that motor neuron undergoes necroptosis by using the spinal cord of the ALS model. Further, these reports suggested that neuronal cell type-specific necroptosis occurs in neurodegenerative diseases. Recent studies have been noted that application of Nec-1 has a protective effect on dopaminergic neurons after treatment of 6-OHDA to PC12 cells and diminished the expression of LC3, involved in autophagy. These results indicated that expression of RIPK1 is high in the Parkinson disease (PD) model [88].

## Necroptosis—therapeutic approach for neurodegenerative disease

The protective and inflammatory effects of necroptosis in pathological condition of neurodegenerative diseases are used to develop effective treatments. In recent times, there are some inhibitors (or compounds) which are used to treat the neurodegenerative diseases such as NEC-1, HDAC inhibitors, and 24(S)-hydroxycholesterol. Nec-1 diminished the activity of RIPK1 by inhibiting its phosphorylation and translocation resulting in disruption of downstream necroptosis signaling [3]. In spite of necroptosis, Nec-1 also inhibits apoptosis and autophagy through activating Akt and mTOR signal pathways after traumatic brain injury (TBI) [100]. Previous studies based on Nec-1 demonstrated that inhibition of RIPK1 blocked the cell death including necroptosis and apoptosis in an animal model of degenerative diseases. Using Nec-1 with zVAD-fmk produced promising treatment effects in neurodegenerative diseases [2], suggesting that there is great complementarily between necroptosis and apoptosis. 24(S)-Hydroxycholesterol (24S-OHC) plays a key role in maintaining cholesterol homeostasis in the brain and considered as a possible biomarker of neurodegenerative diseases [101]. Ester form of 24S-OHC inhibits amyloid-β production at physiological concentration while at high concentration it induces non-apoptotic programmed cell death in neuronal cells with low expression of caspase-8; hence, controlling the level of 24S-OHC may prevent onset of progressive neurodegenerative diseases [101]. Histone modifications have a great impact on epigenetic modulation of transcription in cells. Acetylation and deacetylation are two important modifications involved in regulation of transcription status of cells. Deacetylation of histone protein results in chromatin condensation and transcriptional inhibition in neurodegenerative diseases including Huntington’s disease and PD [102–104], suggesting that HDAC is closely related with necroptosis in contrast to apoptosis. Suberoylanilide hydroxamic acid (SAHA) is a most appropriate necroptotic inhibitor which could protect cells from necroptosis [97] by enhancing the expression of cFLIPL, NF-κB, and P38 while inactivating the JNK [49].

ALS is a rare progressive neurodegenerative disease which affects the nerve of the brain and spinal cord leading to weakening/hardening of muscles on onset of age, and there is no effective treatment to reverse the natural course of ALS by far. Ablation in function of optineurin (OPTN) [64] and superoxide dismutase-1 (SOD1) [99] resulted in pathogenesis of ALS. Mutation in OPTN gene actively initiates the RIPK1-dependent signaling resulting in progressive axonal degeneration and demyelination in ALS patients. Interestingly, axonal degeneration and motor dysfunction have been found to reduce in SOD1 G93A transgenic mice on silencing of RIPK3 or Nec-1 (7–Cl–O–Nec-1) stimulation [89] and suggested that astrocytes in motor neuron of ALS patient stimulate necroptosis in RIPK1/MLKL-dependent manner. Degeneration of motor neuron in ALS patient was found to be dead due to necroptosis in in vitro model of human adult primary sporadic ALS [99]. Treatment of ALS patient with necrosulfonamide (NSA) or RIPK1 knockdown resulted in survival of neighboring motor neuron [89, 99]. In necroptotic astrocytes, expression of TLR and myeloid differentiation primary response gene 88 (MyD88) has been found to be upregulated during inflammatory responses. Recently, it was found that activated astrocytes undergo transformation into M1 microglia/macrophages mediated by TLR/MyD88 signaling cascade [92]. Pirooznia et al. [105] have shown that human adult astrocytes of familial and sporadic ALS secreted pro-inflammatory cytokines resulting in the death of neighboring motor neurons through necroptosis. Further, these studies indicated that necroptosis might be a novel target for the treatment of ALS. In spite of this, Nec-1 also ameliorates another neurodegenerative disease (Table 1).

**Table 1:** Role of Nec-1 in neurodegenerative diseases

S. no.	Neurodegenerative disease	Role of Nec-1	References
1	Alzheimer’s disease	Nec-1 promotes the neural cell death and improves neurobehavior in Al-treated mice model. Nec-1 also ameliorates cognitive dysfunction, significantly reducing the level of Aβ and tau in APP/PS1 mice model.	[106, 107]
2	Parkinson’s disease	Nec-1 enhanced the cell survival in 6-OHDA-treated PC12 cells	[108]
3	Huntington’s disease	Nec-1 increased survival rate in ST14 8plx cells	[100]

## Conclusion

Necroptosis, a new type of cell death pathway, and its molecular players contribute to embryonic and postnatal development. In spite of all this, they also participate in tissue homeostasis and in development of various kinds of pathological conditions. Necroptosis is an emerging field closely related to apoptosis and targeting RIPK3 and RIPK1 may help to overcome therapeutic hurdles in the treatment of inflammatory and neurodegenerative diseases. The process of inflammation is a highly complicated process which results due to coordination of different players of the immune system. Necroptosis affects different cell population of the immune system in a temporal and spatial manner. Hence, it is necessary to unveil the molecular mechanism through which necroptosis process regulates the phenotype of immune cells. Further, unrevealing the immunostimulatory mechanisms from apoptosis to necroptosis will be helpful to understand necroptosis. In spite of necroptosis, RIPK3/RIPK1 also mediates no-necroptosis pathway; hence, carefully dissecting these pathways will provide better targets for therapeutic approaches in the nearby future.
